# Role of Octreotide in Menetrier’s Disease: Case Report and Review of Literature

**DOI:** 10.7759/cureus.11515

**Published:** 2020-11-16

**Authors:** Aadil Khan, Anuj Chhaparia, Muhammad B Hammami, Christine Hachem

**Affiliations:** 1 Internal Medicine: Gastroenterology, Saint Louis University School of Medicine, St. Louis, USA; 2 Internal Medicine, Saint Louis University School of Medicine, St. Louis, USA; 3 Internal Medicine: Gastroenterology, University of California Riverside, Riverside, USA

**Keywords:** menetrier's disease, octreotide, gastric adenoma, juvenile polyposis syndrome

## Abstract

Menetrier’s disease (MD) is a rare disease characterized macroscopically by gastric rugae thickening and microscopically by foveolar hyperplasia with glandular atrophy, resulting in luminal protein loss. Different treatment strategies, including antibiotics, prednisone, octreotide, and monoclonal antibodies, have yielded varying degrees of success. Here, we present a rare complication of MD with a gastric outlet obstruction from a large adenoma. However, prior to this complication, dramatic clinical and laboratory improvements were observed after 12 months of treatment with subcutaneous octreotide. We also present a review of the literature for the role of octreotide in the treatment of MD.

## Introduction

Menetrier’s disease (MD) is a protein-losing enteropathy (PLE). The uniting factor of PLE conditions is the excessive sequestration of serum protein into the gut lumen. This leads to loss of serum protein [1]. MD presents as enlarged rugae due to hyperplastic mucous cells in the stomach wall. Because the rugae are enlarged, there is an abnormally high number of mucous cells, which release protein-containing mucous into the stomach. As a result, hypoproteinemia ensues, as hypersecretion of mucous into the gastrointestinal tract depletes plasma proteins. Patients with this disorder present with a plethora of symptoms and signs, including nausea, vomiting, abdominal pain, edema in peripheral tissues, anemia, hypoalbuminemia, and hypochlorhydria.

While the exact etiology of MD is unknown, there have been experiments implicating increased signaling of epidermal growth factor-receptor (EGF-R) through transforming growth factor ɑ (TGF-ɑ) [2]. MD has also been shown to be associated with cytomegalovirus and *Helicobacter pylori* as well as other infections, but there are also many cases in which these microorganisms have not been detected [3].

Treatment of MD through drugs has yielded inconsistent or mixed results. Several pharmacological agents, including prednisone, antibiotics, specific *H. pylori* treatment, and non-steroidal anti-inflammatory drugs have shown variable success and no universal therapy is established [4]. Cetuximab has more recently been cited as an effective therapy, but it is not a cure. [5] Gastrectomy remains the treatment of choice for cases refractory to medical treatment [6]. In this case, we present a patient with MD which was successfully managed using octreotide, a somatostatin analog.

## Case presentation

**Figure 1:**
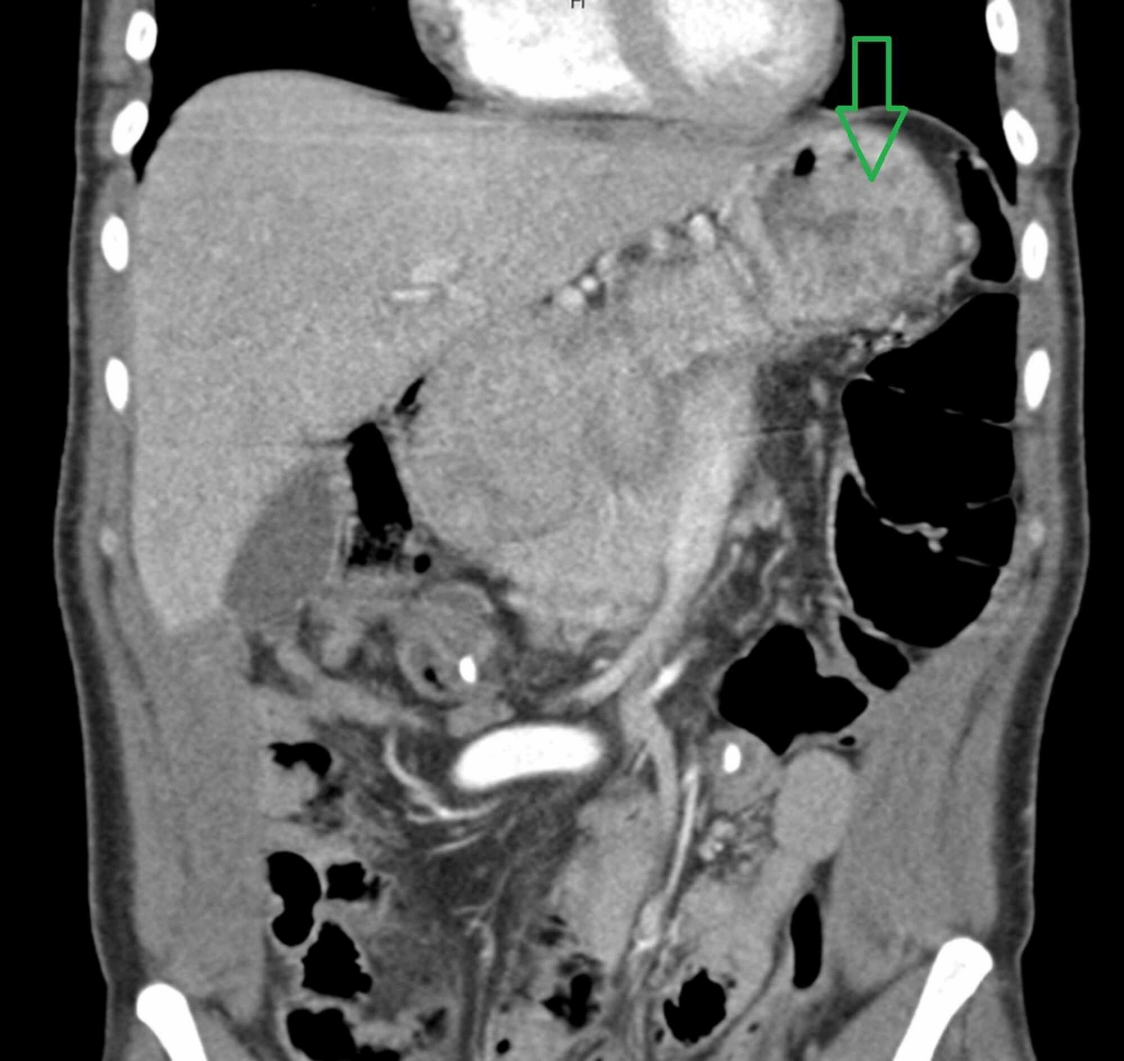
Coronal section showing mass-like thickening of the gastric wall.

**Figure 2:**
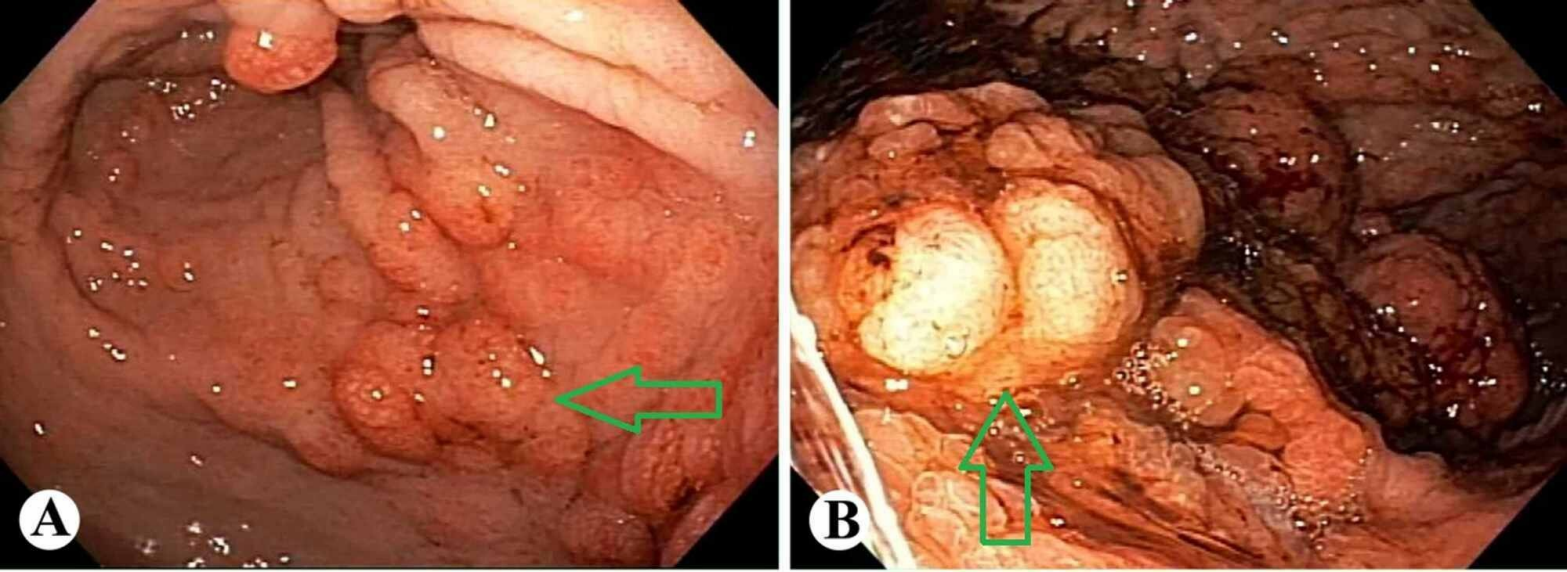
Endoscopic appearance of the stomach showing edematous mucosa and polypoid changes of the gastric fundus (A) and body (B).

**Figure 3:**
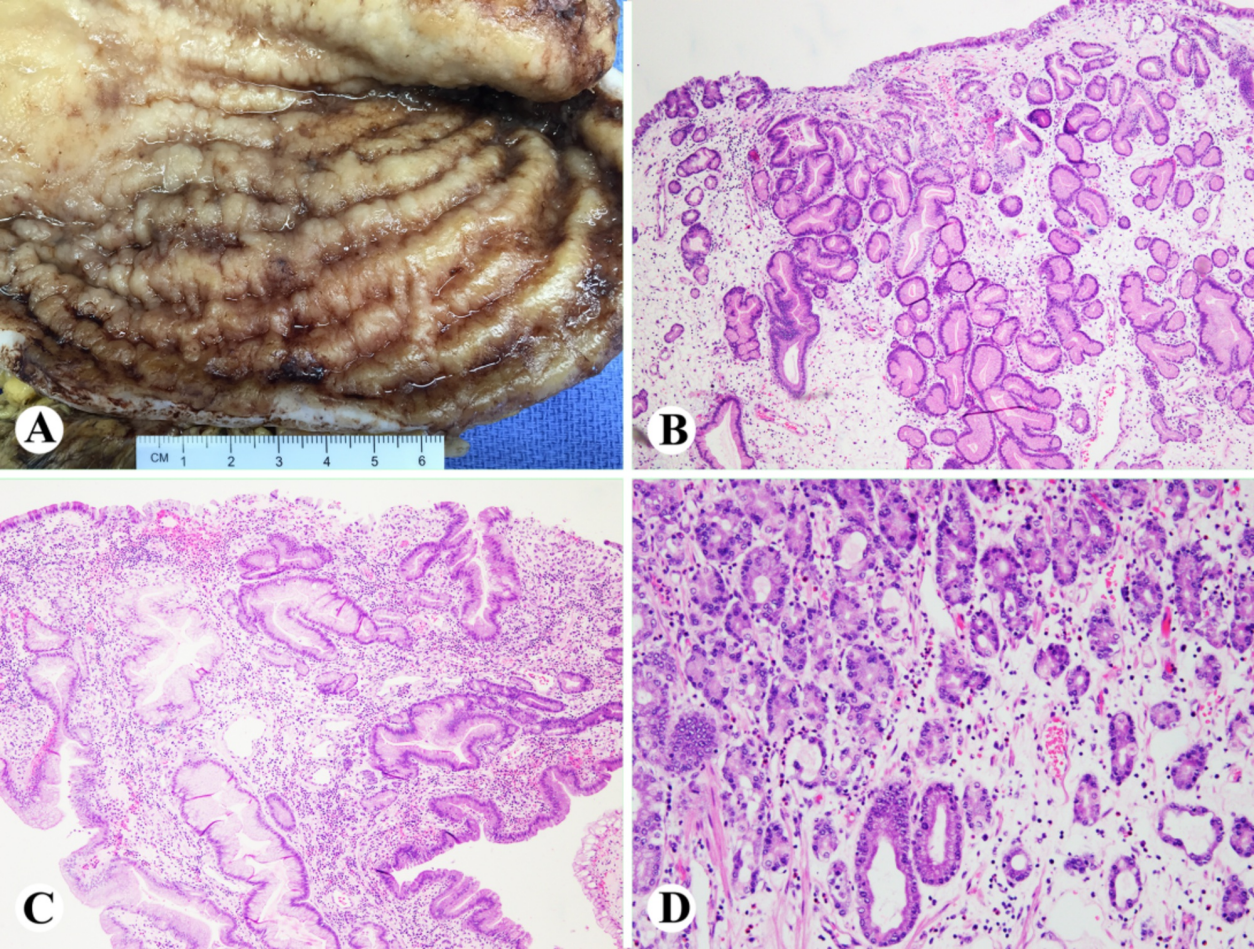
Grossly, hypertrophied gastric mucosa (A); histologically, gastric body and fundal mucosa with edematous, mildly inflamed lamina propria with hyperplastic, tortuous and dilated foveolar glands, and loss of parietal cells (B-D).

A 52-year-old African American man presented for evaluation of fatigue and anemia. He was noted to have a hemoglobin of 6 g/dL with a mean corpuscular volume (MCV) of 58 μm3. On initial imaging, he exhibited what appeared to be a large gastric mass or a thickened fold (Figure 1). An esophagogastroduodenoscopy (EGD) demonstrated large gastric folds with increased thickness (Figure 2). Gastric biopsies were performed and revealed dilated hyperplastic foveolar glands with stromal edema, characteristic of MD (Figure 3). In addition, lab values were consistent with ferritin of 8 ng/ml, iron of < 5 mcg/dL, and an albumin of 0.9 g/dL (Table 1). A stool alpha-1-antitrypsin showed high clearance (>59mL/day). The diagnosis of MD was made using the pathognomonic endoscopic and histologic findings in the setting of hypoalbuminemia, iron deficiency anemia and positive 24-hour alpha-1-antitrypsin clearance test. No microorganisms, including *H. Pylori *and CMV, were identified.

**Table 1:** Effect of octreotide treatment on laboratory values in a patient with Menetrier's disease.

	Day 1 of octreotide treatment	Six months into octreotide treatment	One year into octreotide treatment
Albumin (g/dL)	0.9	2.8	2.9
Hemoglobin (g/dL)	6.0	14.4	14.4
Iron (mcg/dL)	<5	116	125
Body Weight (lbs)	164	159	180
Ferritin (ng/mL)	8	37	19
Alpha-1-Antitrypsin (mg/dL)	220	NR	370

The patient was started on 100µg twice daily of subcutaneous octreotide for seven months and then transitioned to 20mg of octreotide long-acting release (LAR) for five months. The entire course of treatment was 12 months, and during that time, his albumin increased from 0.9g/dL to 2.9g/dL; his anemia and iron deficiency returned to normal values (Table 1). He also regained significant muscle mass that he had previously lost before treatment (Table 1).

**Figure 4:**
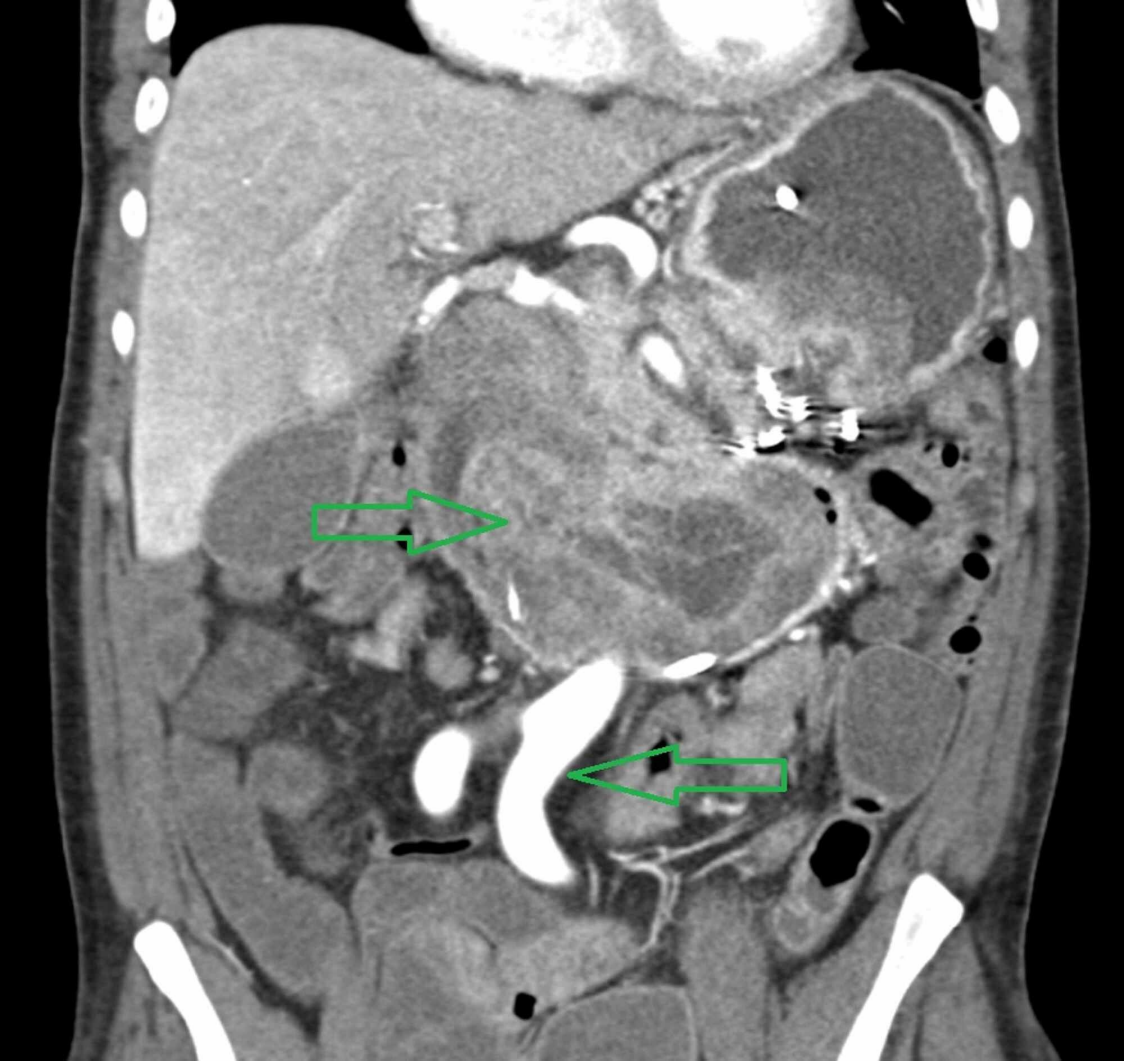
Representative image of large soft tissue density mass filling and markedly distending the duodenum and is favored to represent a pedunculated mass arising from gastric antrum that has migrated into the duodenum.

**Figure 5:**
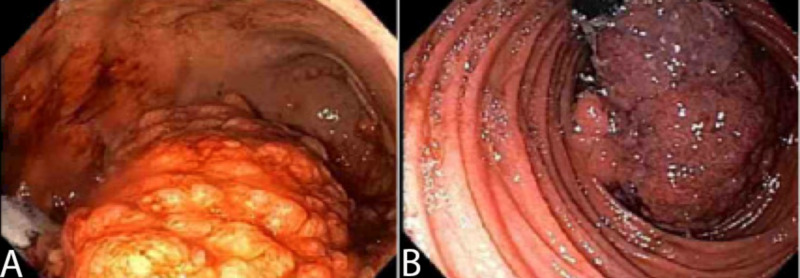
Endoscopic appearance of stomach showing large, fungating, partially circumferential mass (A) and retroflexed view from fourth portion of duodenum showing large nearly obstructive duodenal mass extending the full range of duodenum (B).

**Figure 6:**
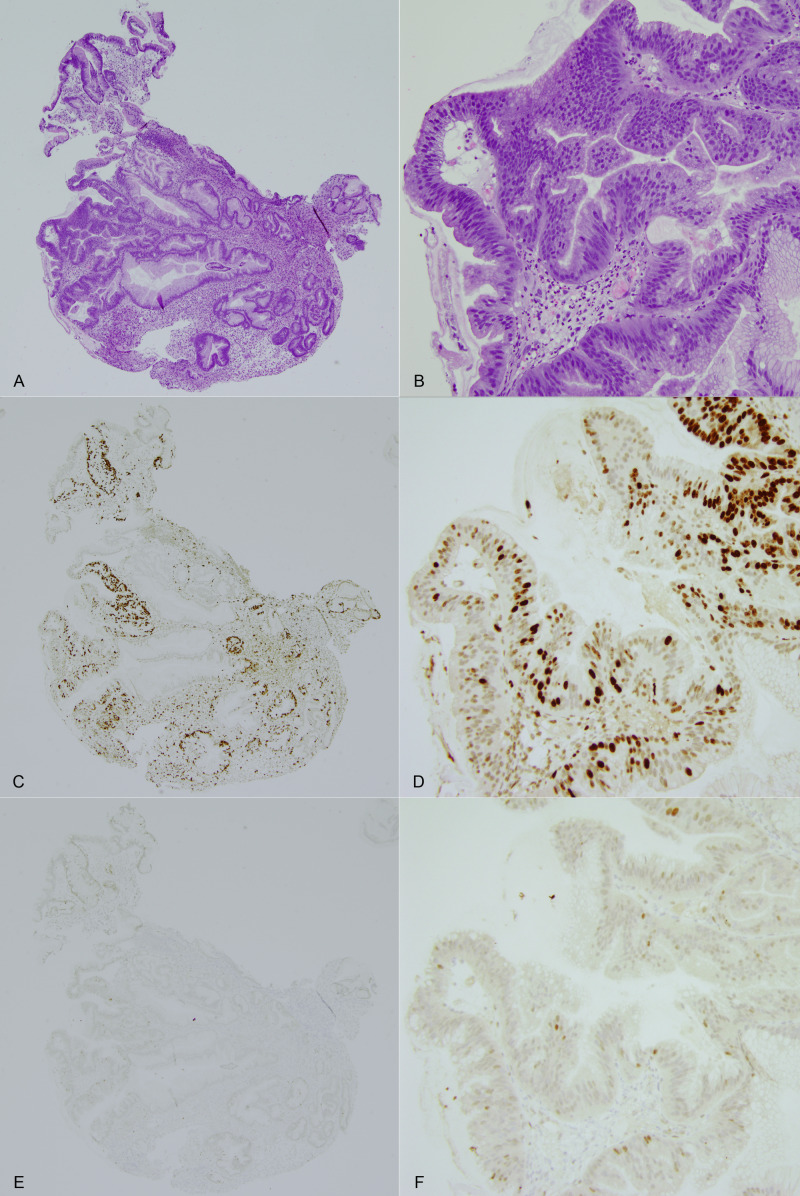
Hematoxylin and eosin-stained sections at 40x and 200x magnification. Hematoxylin and eosin-stained sections at 40x and 200x magnification show a gastric adenoma in the left half of the specimen, with foveolar epithelium in the adjacent right half. Goblet cells are seen at lower magnification (A) in the top left corner (A-B). Immunohistochemical staining for MIB-1 (Ki-67) at 40x and 200x magnification shows increased proliferative activity in the adenoma portion, with minimal activity in the adjacent foveolar epithelium (C-D). Immunohistochemical staining for p53 at 40x and 200x, which is used to highlight high-grade dysplasia, shows rare positive cells in the adenoma portion of the specimen and no staining in the adjacent epithelium (E-F).

At the end of his 12-month treatment with octreotide, he developed a gastric outlet obstruction (Figure 4). He underwent an EGD which showed a large gastric polyp extending into the duodenum resulting in near complete obliteration of duodenal lumen (Figure 5). Pathology was consistent with gastric adenoma (Figure 6). The patient required total gastrectomy with Roux-en-Y gastrojejunostomy for gastric outlet obstruction. He recovered fully from the surgery and continued to do well up to three years of follow up. Around this time, the patient was also diagnosed with juvenile polyposis syndrome (JPS). He had a history of multiple colonic tubulovillous adenomas with dysplastic changes and genetic screening revealed a SMAD4 c153dupA mutation. He is enrolled in a cancer screening program and is receiving regular colonoscopies, EGDs, video capsule endoscopies and endoscopic ultrasounds for pancreatic cancer.

## Discussion

This patient benefited from octreotide treatment as aberrant lab values normalized, gastric mucosa regressed, and clinical symptoms subsided after completing 12 months of octreotide treatment. This is consistent with a few other published cases of MD treated with octreotide (Table 2) [7-13]. The majority of these patients were males between the age group of 30-75 years and underwent treatment ranging from 3-15 months. The treatment protocol utilized in these cases was either 20mg octreotide LAR every 28 days alone or 100-500µg octreotide daily followed by 20mg of octreotide LAR at every 28-day dosing. In our patient, there was a trend towards normalization of laboratory values and improvement in clinical symptoms within 6-12 months of octreotide treatment. Unfortunately, despite improved symptoms and signs, he developed intestinal obstruction from a gastric adenoma requiring gastrectomy. Given the timing of the adenoma appearance, one may suggest a relationship between either MD or octreotide treatment and adenoma. In addition, there is currently a hypothesis that MD is a premalignant condition. According to Hsu et al. [14], about 6-10% of patients with MD later develop gastric adenocarcinoma. While the exact etiology of this adenoma is unknown, JPS may have played a larger role than the MD or octreotide therapy.

**Table 2:** Published cases of Menetrier’s disease treated with octreotide.

Author/Year	Age (Year)/Sex	Method of Diagnosis	Treatment	Duration of Treatment	Outcome of treatment
Chebli et al. 2017 [7]	54/Male	Elevated alpha-1 antitrypsin, endoscopy and subsequent biopsy	20mg octreotide LAR once every 28 days w/ high protein diet	3 months	Asymptomatic, increased albumin
Xiong and Gong 2016 [8]	56/Male	Endoscopy and subsequent biopsy	20mg octreotide LAR once every 28 days	3 months	Treatment started after symptoms and lab values stabilized, little/no regression of gastric mucosa
Nardo et al. 2012 [9]	4/Male	Endoscopy and subsequent biopsy	50µg octreotide twice a day followed by Octreotide LAR 5mg once every 28 days	15 months	Asymptomatic, normalized hemoglobin, slight regression of gastric mucosa
Rothenberg et al. 2009 [10]	75/Male	Endoscopy and subsequent biopsy	150µg octreotide every 8 hrs followed by 20mg Octreotide LAR	3 months	Asymptomatic, normalized albumin, normal gastric mucosa
Gadour et al. 2005 [11]	31/Female	Endoscopy and subsequent biopsy	200mg octreotide daily	6 months	Asymptomatic, normalized albumin, normal gastric mucosa
Green and Branch 2004 [12]	29/Male	Elevated alpha-1 antitrypsin, endoscopy and subsequent biopsy	100µg 3 times per day followed by octreotide LAR once every 28 days	9 months	Asymptomatic, normal albumin and alpha-1 antitrypsin
Yeaton and Frierson 1993 [13]	47/Male	Endoscopy, albumin stool studies	100µg octreotide	12 months	Asymptomatic, urgent gastrectomy not necessary

The exact pathophysiology of MD is still evolving with various potential triggers, but TGF-ɑ and EGF-R seem to play a pivotal role. TGF-ɑ is a signaling molecule ordinarily present in the stomach and is known to be one of many ligands of EGF-R. In vitro studies suggest that TGF-ɑ causes proliferation of gastric epithelial cells, increase in mucin production, atrophy of oxyntic cells, and reduction in acid production. In mouse models, upregulation of TGF-ɑ results in a phenotype similar to MD [15]. It is also plausible to suggest increased levels of TGF-ɑ may play a role in the development of adenoma, as it increases the proliferation of gastric epithelial cells. However, current mouse models have not demonstrated a direct link between TGF-ɑ and gastric adenoma [16]. Using the elevated TGF-ɑ hypothesis, it is difficult to directly link the development of an adenoma in our patient with his MD. 

The pharmacology of octreotide, a somatostatin analog, in MD is multifactorial. On a molecular level, somatostatin has been postulated to affect TGF-ɑ/EGF-R signaling at multiple points. First, somatostatin reduces the number of EGF-R on the cell surface, resulting in decreased TGF-ɑ/EGF-R signaling [17]. Second, certain downstream elements (e.g., mitogen-activated protein kinase [MAPK]) are shared between EGF-R and the somatostatin receptor. By activating somatostatin receptors, octreotide may weaken EGF-R signaling, which is increased in MD via these downstream elements [18]. Third, a subtype of somatostatin receptor (SST5) may heterodimerize with EGF-R after binding with octreotide, resulting in an altered interaction between TGF-ɑ and EGF-R [19]. Given the multiple mechanisms by which octreotide inhibits the activity of EGF-R, it is unlikely that octreotide injections played a role in the pathogenesis of the adenoma.

In contrast to MD and octreotide treatment, our patient’s JPS (SMAD4 mutation) was more likely to have led to the development of gastric adenoma. SMAD4 is a tumor suppressor gene involved in regulating cell growth and differentiation. Not only SMAD4 mutation implicated in JPS, but it is also linked with various dysplasias and malignancies, including gastric adenomas [20]. Therefore, this mutation in our patient may explain the sudden appearance of the large gastric adenoma.

## Conclusions

In conclusion, MD is a rare disease in which the treatment guidelines are not well established. The available literature on the use of octreotide in MD is limited, and thus our case report further strengthens its position among available treatment options. We showed that octreotide could provide meaningful clinical and laboratory improvement in MD, as well as possibly delay the need for gastrectomy. Furthermore, our case also highlights a unique presentation of MD in the form of gastric outlet obstruction secondary to gastric adenoma, which may be explained by underlying JPS with SMAD4 mutation.
